# Effects of family-integrated emotion regulation group therapy on non-suicidal self-injury among adolescents with depressive disorders: A randomized controlled follow-up study

**DOI:** 10.1192/j.eurpsy.2026.10201

**Published:** 2026-07-31

**Authors:** N. Du, X.-B. He, X.-J. Nie, Z.-S. Huan, Y. Xiao

**Affiliations:** 1 The Clinical Hospital of Chengdu Brain Science Institute, Chengdu; 2 Chongqing Mental Health Center, Chongqing; 3 Guizhou Moutai Hospital, Guizhou, China

## Abstract

**Introduction:**

In recent years, the prevalence of Non-suicidal self-injury (NSSI) among adolescents has been increasing sharply. Although NSSI may appear less severe than suicidal behavior, it significantly increases the likelihood of future suicide attempts. However, available treatment methods for NSSI are limited. Emotion Regulation Group Therapy (ERGT) has proven effective in teaching adolescents adaptive emotional strategies and reducing NSSI behaviors. However, the role of family relationships in improving outcomes for adolescents with NSSI is also essential.

**Objectives:**

Therefore, we designed the present randomized controlled trial with the following objectives: (1) to evaluate the efficacy of a therapist-delivered, family-integrated ERGT program developed by our team for adolescents with depressive disorders and NSSI; (2) to determine whether this integrated model yields superior and more sustained outcomes compared to traditional ERGT approaches; and (3) to explore potential cognitive and autonomic nervous system changes associated with the intervention.

**Methods:**

This study enrolled adolescents (aged 12–18 years) diagnosed with depression and recurrent episodes of NSSI (n=73). They were randomly assigned to two groups: the trial group (n=36) which received family-integrated ERGT; and the control group (n=37) which received ERGT alone. Data on NSSI behavior, depressive symptoms, and impulsivity were collected using questionnaires, including the Adolescent Non-suicidal Self-injury Assessment Questionnaire (ANSSIQ), the Self-Rating Depression Scale (SDS), and Barratt’s Impulsivity Scale (BIS). All questionnaires were administered at five time points: baseline, immediately post-intervention, and at the 1-month, 3-month, and 6-month follow-ups. The values of heart rate variability (HRV) and event-related potential (ERP) were also collected before and after the intervention.

**Results:**

Immediately post-intervention, no significant differences were observed between the two groups in ANSSIQ, BIS, and SDS scores (P>0.05). However, at all follow-ups, the trial group demonstrated significantly greater reductions in all three measures (P<0.05), with a more pronounced decline over time (see image 1,2,3 for details). Results showed lower N2, P3a, and P3b amplitudes and higher HRV in the trial group post-intervention, with no significant changes in the control group.

**Image 1:**
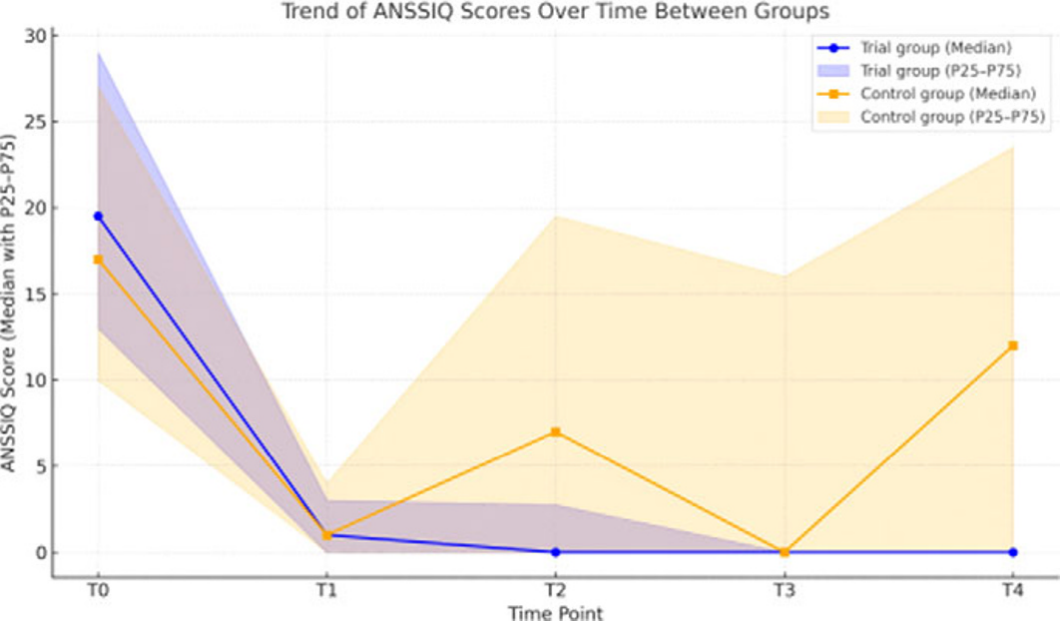
Image 1: Long description.

**Image 2:**
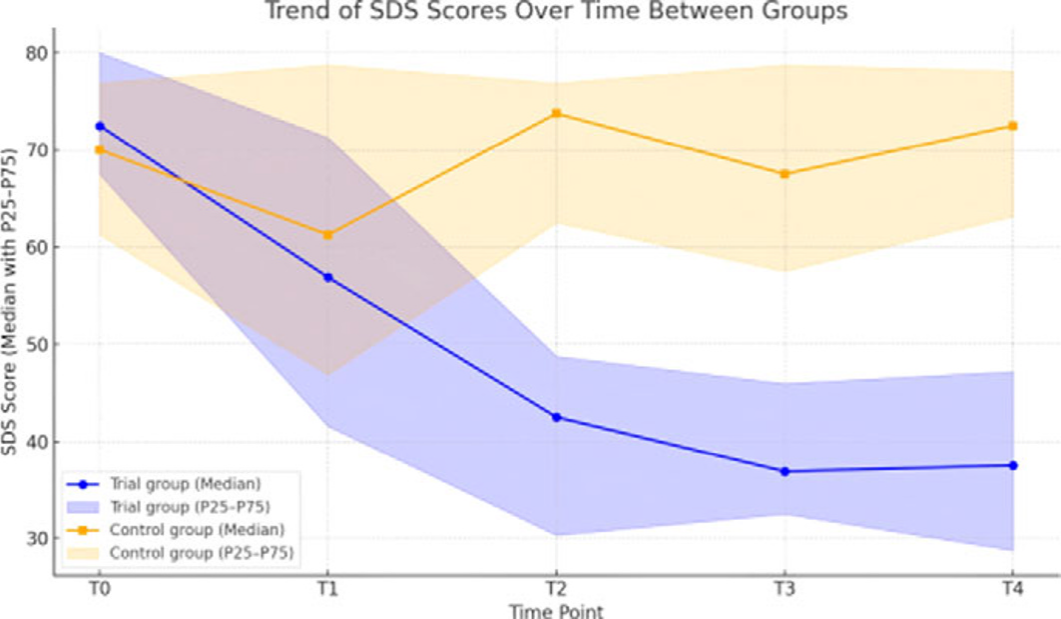
Image 2: Long description.

**Image 3:**
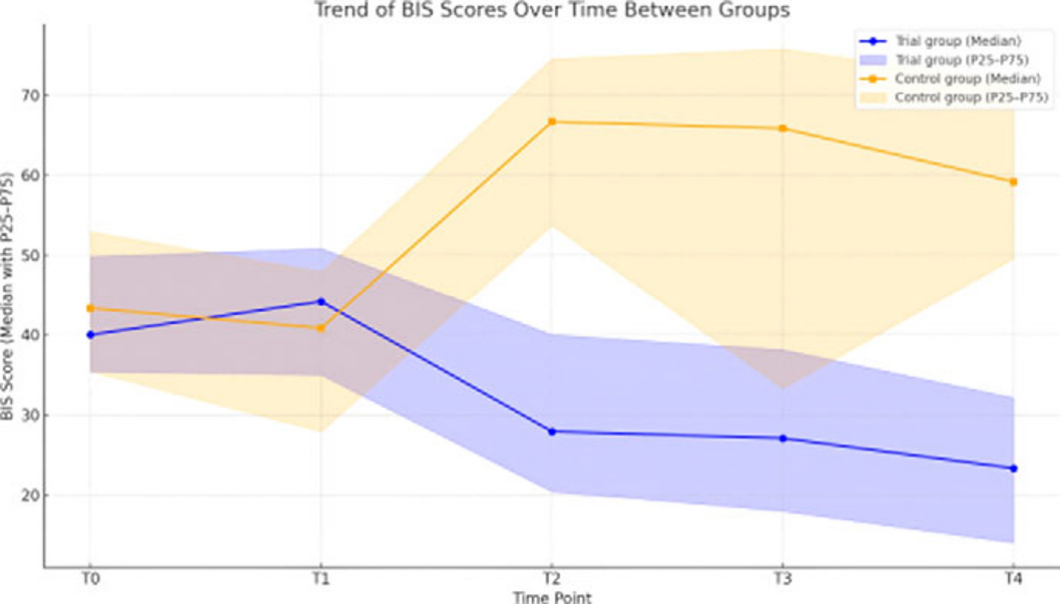
Image 3: Long description.

**Conclusions:**

Our findings suggest that family integration may enhance the long-term efficacy of ERGT in treating adolescent NSSI and associated emotional and cognitive dysregulation, supporting its clinical applicability.

**Disclosure of Interest:**

None Declared

## Long descriptions

### Image 1: Long description

The x-axis is labeled Time Point with five intervals: T 0, T 1, T 2, T 3, and T 4. The y-axis is labeled A N S S I Q Score (Median with P 25-P 75) ranging from 0 to 30.

* Trial group (Median): Represented by a blue line with circular markers. It starts at approximately 19.5 at T 0, drops sharply to 1 at T 1, and then remains at 0 for T 2, T 3, and T 4. The shaded blue area (P 25-P 75) narrows significantly after T 0.

* Control group (Median): Represented by an orange line with square markers. It starts at 17 at T 0, drops to 1 at T 1, rises to 7 at T 2, drops back to 0 at T 3, and rises again to 12 at T 4. The shaded orange area (P 25-P 75) is much wider than the trial group from T 2 onwards, indicating higher variability.

A legend in the top right corner identifies the blue line as Trial group (Median), the light blue shaded area as Trial group (P 25-P 75), the orange line as Control group (Median), and the light orange shaded area as Control group (P 25-P 75).

Navigate back to Image 1:.

### Image 2: Long description

The x-axis is labeled Time Point with five intervals from T 0 to T 4. The y-axis is labeled S D S Score Median with P 25-P 75 and ranges from 30 to 80.

* Trial group (Median): Represented by a blue line with circular markers. It starts at approximately 72 at T 0 and shows a steep linear decrease to 57 at T 1 and 42 at T 2. It then levels off with a slight downward trend to 37 at T 3 and 38 at T 4. A light blue shaded area represents the P 25 to P 75 range, which narrows as the score decreases.

* Control group (Median): Represented by an orange line with square markers. It starts at 70 at T 0, dips to 61 at T 1, then rises to a peak of 74 at T 2. It drops to 67 at T 3 and rises again to 72 at T 4. A light orange shaded area represents the P 25 to P 75 range, which remains relatively wide and consistent throughout the duration.

The two groups begin at similar scores at T 0, but the Trial group shows a significant overall reduction while the Control group fluctuates at a high score level.

Navigate back to Image 2:.

### Image 3: Long description

The x-axis is labeled Time Point with five intervals: T 0, T 1, T 2, T 3, and T 4. The y-axis is labeled B I S Score (Median with P 25-P 75) ranging from 20 to 70. A legend in the top right identifies two groups:

* Trial group (Median): Blue line with circular markers.

* Trial group (P 25-P 75): Light blue shaded area representing the interquartile range.

* Control group (Median): Orange line with square markers.

* Control group (P 25-P 75): Light orange shaded area representing the interquartile range.

Data Trends:

* At T 0, both groups start near a score of 40 to 43 with overlapping shaded regions.

* At T 1, the Trial group rises slightly to 44 while the Control group dips to 41.

* At T 2, a significant divergence occurs. The Control group median sharpens upward to approximately 67, while the Trial group median drops to 28.

* At T 3, the Control group remains high at 66, while the Trial group stays low at 27.

* At T 4, the Control group decreases to 59, and the Trial group continues a slight downward trend to 23.

The shaded regions show that the Control group has a much wider variance from T 2 to T 4 compared to the Trial group.

Navigate back to Image 3:.

